# Optimization Strategy for Parks and Green Spaces in Shenyang City: Improving the Supply Quality and Accessibility

**DOI:** 10.3390/ijerph19084443

**Published:** 2022-04-07

**Authors:** Wen Wu, Kewei Ding

**Affiliations:** Liaoning Provincial Key Laboratory of Urban and Architectural Digital Technology, JangHo Architecture College, Northeastern University, Shenyang 110819, China; 20185758@stu.neu.edu.cn

**Keywords:** urban green space accessibility, service capacity, landscape pattern index, network analysis

## Abstract

In this study, we evaluated the supply quality of parks and green spaces within the Third Ring Road area in Shenyang city by combining a landscape pattern index analysis with a principal component analysis. Moreover, a network analysis based on the ArcGIS platform was used to measure the accessibility of parks and green spaces. The research results showed that the overall supply quality of parks and green spaces (−9.55) must be improved. The supply quality levels of the four analyzed park types could be ranked as follows: garden parks (118.00) > community parks (73.67) > comprehensive parks (−16.64) > specific parks (−32.17). Among the analyzed recreation parks, the accessibility of daily recreation parks was poor, while the overall service efficiency of weekly recreation parks was better, except in a few regions. These research results can provide suggestions for future green space planning in Shenyang city. In addition, from the perspective of landscape patterns, studying the service quality of parks and green spaces can provide new ideas for further research on accessibility.

## 1. Introduction

The rapid development of megacities in China has resulted from expanding economic growth and industrial developments [1]. The speed of development has caused many weaknesses in urban ecosystems [2,3]. Urban ecosystem services help shape resilient and energetic cities in accordance with the concept of sustainable development [4]. Ecosystem service assessments are used to evaluate the benefits to residents from ecosystems [5].

Urban green spaces are an important part of urban ecosystems. Many studies have shown that urban green spaces have positive influences, such as adjusting the local climate [6], reducing noise and air pollution [7,8], coordinating urban spaces and satisfying the daily leisure needs of residents [9,10]. Green spaces provide vital ecosystem services for both sustainable cities and human beings [11]. The recreational services supplied by green spaces improve the welfare of residents [12].

As living standards have improved, citizens have developed higher standards for the quality of their living environments. Thus, people not only focus on the quantity of urban parks but also pay more attention to the supply quality and accessibility of these parks. In urban construction, static indexes such as the urban green space ratio and the per capita park area are typically used to measure the construction standard of urban parks, while the supply quality is considered relatively less [13]. Green space fragmentation is an important aspect of the quantitative evaluations of the quality of green spaces, and ecological and social service functions are highly correlated with this degree of fragmentation. Ensuring the rational arrangement of urban parks can contribute to maximizing the service effect of urban parks in limited urban spaces [14].

Accessibility is one of the main indexes used to evaluate the supply quality and service capability of urban parks. Hansen first proposed the concept of accessibility and defined it as the interaction opportunity between nodes in a traffic network [15]. Thereafter, accessibility was widely used in various fields, such as research regarding public service facilities [16,17,18,19], disadvantaged groups [20,21,22,23] and social equity [24,25,26,27]. Landscape accessibility reflects the relative difficulty of access from any point in the landscape space [28]. The methods used to measure green space accessibility mainly include simple buffering [29,30,31,32,33], the minimum distance method [34,35,36], network analyses [37,38] and the gravity model method [39,40,41]. In simple buffering, the Euclidean distance from the origin point to the center point is used as an analysis index, and the result is a virtual height [42]. The shortcoming of the minimum distance method is that it uses the nearest linear distance to calculate accessibility [43]. Therefore, aggregation errors are less considered in this method [44]. The disadvantage of the gravity model method is that no unified standards exist regarding the classification of urban parks, which increases the difficulty of interpreting the results [45].

Network analyses are abstract simulations of a traffic network and are used to study the flow situation among different resources in the network [46]. This method can simulate traffic situations based on realistic urban roads in research on urban park accessibility. Moreover, considering different travel mode selections and combinations can improve the accuracy and reliability of the results [47,48]. Hence, the main objectives of this study are as follows: (1) to obtain the spatial distribution of urban park green space in Shenyang, China; (2) to determine the supply quality of different types of urban park green space; and (3) to evaluate the accessibility of urban parks by applying a network analysis, thus providing suggestions for the construction of urban parks.

## 2. Study Area and Methods

### 2.1. Overview of the Study Area

Shenyang city is located in the midlands of Liaoning Province (123°18′~123°48′ E, 41°36′~41°57′ N). Shenyang is the provincial capital city of Liaoning Province and is also the political, economic and cultural center of northeast China. The study area is located within the Third Ring Road of the city, covering 455 km^2^ of territorial area. According to the information released by the National Bureau of Statistics in September 2021, Shenyang is considered a megacity. In recent years, large-scale urban internal reforms and urban renewal have been carried out in the Tiexi district and Hunnan new district. The intense changes stemming from the development of the city and industry [49,50] underline the importance and relevance of research on urban parks in the main urban area of the Third Ring Road of Shenyang as city construction moves from incremental expansion to stock optimization.

### 2.2. Data Sources and Pretreatment

**Figure 1:**
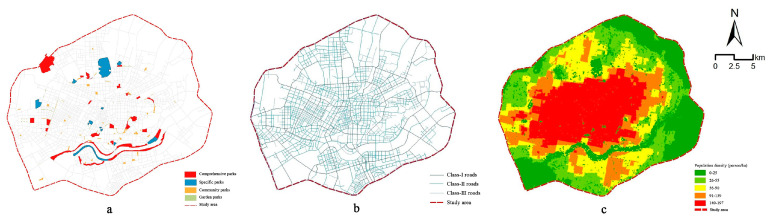
(**a**) The distribution of urban parks within the Third Ring Road of Shenyang; (**b**) the road network within the Third Ring Road of Shenyang; (**c**) the population density distribution within the Third Ring Road of Shenyang.

The data utilized in this study mainly included an administrative division map of Shenyang and satellite remote data of Shenyang collected in 2018 (at a spatial resolution of 0.45 m). Urban parks were visually interpreted and extracted from these data according to the Shenyang City Master Plan (2011–2020) and the classification standard of urban green space (CJJT85-2017) and analyzed parks included comprehensive parks, special parks, community parks and garden parks. Under the current classification standard of urban green space, no definite standard is set for garden parks. Referring to the study of Wu and Wang (2021) [51], an urban public open space with an area of more than 0.1 hectares and a width of more than 15 m is an important urban recreational space. Thus, we specified the classification standard of garden parks using this definition. A database of urban parks (Figure 1a) was built by recording the name, area and category of each park. Table 1 shows the quantity of parks in the study area. The coordinate data of urban park gates were obtained through field investigations and the Baidu map. The centroid of each park was used to replace independent gates among the 15 urban parks without gates [52], and a database of park entrances and exits was also eventually built.

Using ArcGIS 10.7 (Environmental Systems Research Institute (ESRI), Redlands, CA, USA), city roads were extracted from remote sensing images, and road vector data were drawn based on the realistic construction of roads in the study area. The average road width was obtained using a field calculator. In reference to the study of Lu et al. (2014), roads were divided into three classes based on width: class-I roads had widths greater than 30 m, class-II roads had widths between 15 and 30 m and class-III roads had widths less than 15 m [52]. The topology tool was used to check the road widths and obtain road network data in the study area (Figure 1b). Residents can reach urban parks through three modes: walking, nonmotor vehicles and motor vehicles. We used 3.6 km/h as the walking speed according to relevant research [53]. A bicycle, a nonmotor vehicle, has a speed of 10 km/h. The motor vehicle speeds were set to 60 km/h on class-I roads, 40 km/h on class-II roads and 20 km/h on class-III roads. Moreover, a 30 s intersection waiting time was uniformly set [54].

Population data were derived from the open population density data set with a 100 m spatial resolution obtained by Ye et al. (2019) using a random forest model based on the inversion of 2018 nighttime light data representing Shenyang city [51,55]. Figure 1c shows the population density distribution in the study area.

### 2.3. Methods

#### 2.3.1. Landscape Pattern Analysis

A landscape pattern can be used to emphasize the combined effect of patch types and the layout effect of the overall landscape [56,57]. Landscape/space indexes are quantitative indicators of the composition and spatial allocation characteristics of different landscape types [58] and can reflect highly condensed landscape pattern information. Thus, these indexes have been widely used in research methods to analyze the relationship between landscape ecology patterns and processes [59]. Based on landscape ecology theories, landscape pattern indexes are typically adopted to quantitatively measure the spatial distribution of urban green spaces and evaluate the landscape quality of urban parks. In this work, first, urban park data were rasterized, and three indicators (area, shape and aggregation) were selected for analysis. The area indicators included the patch density (PD), landscape patch index (LPI) and landscape shape index (LSI); the shape indicators included the weighted patch area size (AREA_AM), average shape index (SHAPE_MN) and division index (DIVISION), and the aggregation indicators included the mesh size (MESH), split index (SPLIT) and aggregation index (AI). Then, the landscape index calculation software Fragstats4.0 was used to calculate the landscape pattern indexes of the four types of parks and the parks as a whole. Finally, the principal component analysis (PCA) method was used to integrate the index results and evaluate the comprehensive supply quality of urban parks.

#### 2.3.2. Principal Component Analysis (PCA)

PCA is the main method used to establish a minimum data set. It is a multivariate statistical analysis method in which a small number of important variables, called the principal components, are selected by linearly transforming multiple variables [60]. PCA integrates the comprehensive indexes of variables with minimum loss of original information [61,62]. The formula for PCA is as follows (Equation (1)):

(1) $$F_{P}=a_{1i}Z_{X1}+a_{2i}Z_{X2}+a_{3i}Z_{X3}+\ldots +a_{pi}Z_{XP}$$

where $a_{1i},\;a_{2i}\ldots ,a_{pi}\left(i=1,2,\ldots ,m\right)$ is the eigenvector corresponding to the eigenvalue of the covariance matrix of X. $Z_{X1},\;Z_{X2}\ldots ,\;Z_{XP}$ is the normalized value of the original variable.

In this work, the evaluation indexes were then nondimensionalized after calculating the landscape pattern index for different urban park categories; then, PCA was implemented, and the principal component linear equation was established by using the eigenvectors containing eigenvalues.

#### 2.3.3. Network Analysis

**Figure 2:**
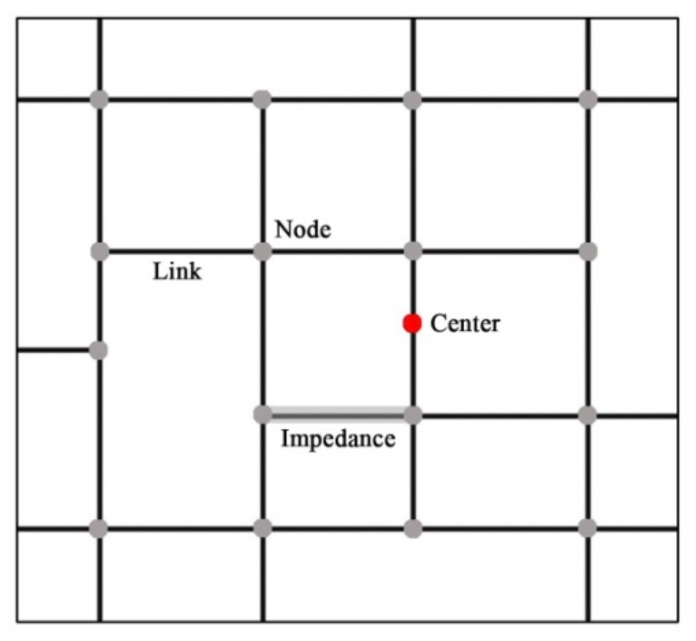
The network analysis diagram.

In a network analysis, the coverage areas of urban parks are calculated with regard to different travel modes under a certain resistance based on the road network. As shown in Figure 2, a complete road network consists of centers, links, nodes and impedances [30]. The center represents the spatial location of a park, which was replaced by the park entrance and exit in this study. The links are city roads, and the nodes represent road intersections. Impedances refer to travel time on the roads, and diverse impedances occur in relation to different roads [63].

#### 2.3.4. Overlay Analysis

The accessible area/population ratio was adopted in this work to evaluate the service efficiency of urban parks [64]. The network analysis method was used to count the accessible areas corresponding to the three travel modes by setting different time thresholds. The results were then superimposed with the population data to obtain the accessible populations. The formulas used to calculate the park green space service efficiency are as follows (Equations (2) and (3)):

(2) $$\mathrm{accessible}\;\mathrm{area}\;\mathrm{ratio}=\frac{\mathrm{accessible}\;\mathrm{area}}{\mathrm{study}\;\mathrm{area}}\times 100\%,$$

(3) $$\mathrm{accessible}\;\mathrm{population}\;\mathrm{ratio}=\frac{\mathrm{accessible}\;\mathrm{population}}{\mathrm{study}\;\mathrm{population}}\times 100\%.$$

## 3. Results

### 3.1. Spatial Distribution of Urban Park Green Space

A total of 170 urban parks were obtained through the extraction and interpretation of remote sensing images, covering a total area of 2123.14 hectares. The study area was dominated by large parks, including comprehensive parks and special parks; these two park types accounted for 87.8% of the total park area. Community parks and garden parks were relatively sparse, especially when considering the number and scale of garden parks. The total area of garden parks was 35.60 hectares, accounting for only 1.7% of the total area. Urban parks were mainly constructed around natural water systems such as the Hun River and artificial water systems such as the Weigong Canal. Because the Hun River, the South Canal and the Xinkai River flow through Heping district, Shenhe district and Hunnan new district, which have high populations, a series of comprehensive parks and community parks have been built along these waterways. Artificial water systems are associated with inevitable environmental weaknesses compared to natural water systems, so along the Weigong Canal, ribbon parks composed primarily of garden parks were built. In comparison, the construction of a comprehensive park is less intensive. From the perspective of the whole study area, urban parks were mainly distributed in the southern region. In the northern part of the study area, with the exception of several large parks, such as Beiling Park and Dingxiang Lake Park, the numbers of community parks and garden parks were lower than those in the south. Park distribution was closely related to water systems distribution. Areas without water systems contained concentrated community parks and garden parks, for which the numbers were not quite sufficient.

### 3.2. Supply Quality of Urban Park Green Space

Table 2 reflects the results of the landscape pattern index analysis. Two principal components were extracted in the PCA. The eigenvalues of these two principal components were 5.498 and 2.837, and the corresponding contribution rates were 61.09 and 28.19%, respectively; the cumulative contribution rate was 89.28%. This indicated that these principal components were highly reliable. The principal component linear equation was established using the following eigenvectors of the two eigenvalues (Equations (4) and (5)):

(4) $$F1=-0.095X_{1}+0.409X_{2}-0.114X_{3}-0.382X_{4}-0.315X_{5}+0.378X_{6}+0.378X_{7}+0.341X_{8}+0.405X_{9},$$

(5) $$F2=0.607X_{1}+0.099X_{2}+0.559X_{3}-0.185X_{4}-0.348X_{5}-0.248X_{6}-0.248X_{7}+0.066X_{8}+0.161X_{9}$$

where $X_{1}\sim X_{9}$ corresponds to the numerical value after the index standardization of the PD, LPI, LSI, AREA_AM, SHAPE_MN, DIVISION, MESH, SPLIT and AI. According to these equations, the corresponding principal component scores of various park types were calculated, and finally, a comprehensive green space supply quality evaluation was carried out. The comprehensive evaluation equation used in this study was as follows (Equation (6)):

(6) E=0.6109×F1+0.2819× F2

where F1 and F2 are the scores of the first and second principal components, respectively.

Table 3 indicates that the comprehensive supply quality scores of the four park types were ranked as follows: garden parks (118.00) > community parks (73.67) > comprehensive parks (−16.64) > specific parks (−32.17). The scores derived for garden parks and community parks were much higher than those obtained for special parks and comprehensive parks. The score obtained for overall parks lay between those of community parks and specific parks but was still low (−9.56) and far from the scores of garden parks and community parks.

### 3.3. Accessibility of Urban Park Green Spaces

**Figure 3:**
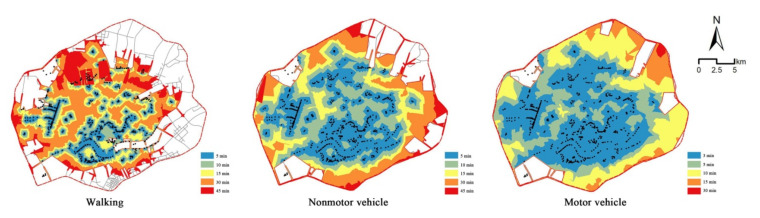
The regions with accessible urban parks under three travel modes.

The accessibility results corresponding to the walking mode (Figure 3) showed that because a series of parks were built along the Hun River and the South Canal, the accessibility of the surrounding regions was high, presenting a state of spatial agglomeration. The number of parks along the Xinkai River was low, so the accessibility in this region was relatively poor. In addition, the accessibility of this region presented a scattered spatial distribution. A number of ribbon parks were built along the Weigong Canal, leading to a linear agglomeration phenomenon, but the accessibility of other areas in the Tiexi district was poor. According to the requirement of “green in 15 min” for urban park construction, accessible areas accounted for only 28.1% of the study area, and accessible populations accounted for 44.7% of the total population in the study area. Thus, more than half of the study area and population did not have access to green spaces. These results indicated that under the walking mode, the current distribution of urban parks had difficulty meeting the demands of city residents.

**Figure 4:**
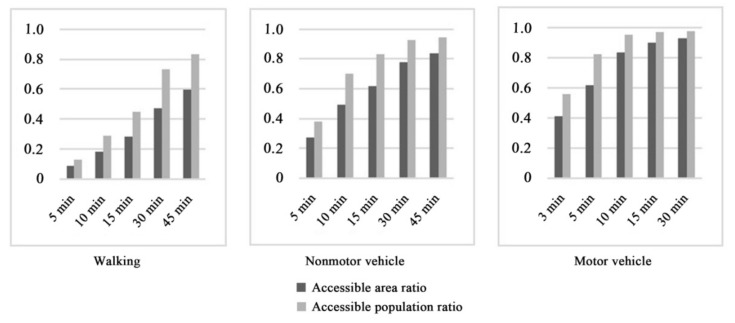
The service efficiency levels of urban parks under three travel modes.

The service efficiency obtained for nonmotor vehicles was better than that obtained for walking, and the accessible area and population were higher compared to the walking mode under the same time thresholds (Figure 4). When the travel time was set to 5 min, the accessible coverage area accounted for 27.2% of the total study area; at 10 min, it accounted for 49.2%, and at 15 min, it accounted for 61.8%. Thirty-eight percent of the total population in the study area could reach green spaces in 5 min; 70.4% in 10 min; and 83.4% in 15 min. The accessible area and population increased fastest in the 5~10 min range. After 10 min, these values showed steady growth trends. The higher speeds of motor vehicles meant their service efficiency level increased greatly compared to the service efficiency levels of walking and nonmotor vehicles. Within 3 min, park-accessible areas accounted for 40.9% of the total study area, and the accessible population accounted for 56.1% of the overall population. The 5 min accessible area comprised 61.7% of the study area and 82.2% of the population. The area reachable in 10 min accounted for 83.5% of the study area and 95.0% of the population. In 15 min, except for some areas that could not be reached because of the incomplete road network, the whole study area was basically covered. Moreover, 96.9% of the population could access urban parks.

When city residents participate in activities in urban parks, they choose different travel modes depending on park types and their specific purposes. Therefore, it was necessary to divide the travel modes according to the different park types to evaluate park accessibility, as this was more in line with the reality of residential park use. The studied parks were divided into daily recreation parks and weekly recreation parks according to the recreation types [64]. Daily recreation parks included community parks and garden parks, while weekly recreational parks included comprehensive parks and special parks [65]. Generally, residents reach daily recreation parks mainly by walking or nonmotor vehicles and access weekly recreation parks by motor vehicles. By considering the actual recreational behaviors of residents, studying park accessibility through different activity circles had more practical significance for the construction of urban parks.

**Figure 5:**
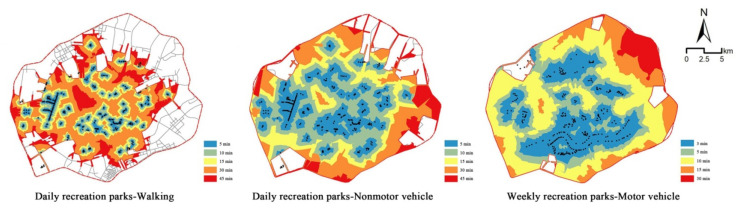
The accessible regions of parks associated with different recreation types.

**Figure 6:**
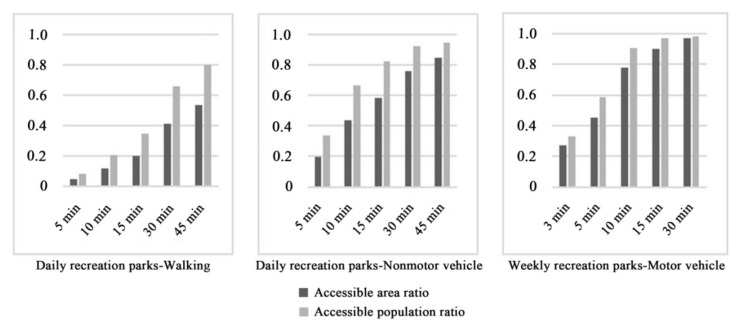
The service efficiency levels of parks associated with different recreation types.

Figure 5 and Figure 6 show the accessibility results obtained for parks associated with different recreation types. The service efficiency of daily recreation parks was poor for the walking mode, except in Shenhe district, Heping district and the areas around the Weigong Canal; the accessibility levels in the other regions were relatively insufficient. At 15 min, the coverage of daily recreation parks comprised only 20.1% of the study area and 35.0% of the urban population. These shares improved greatly for the nonmotor vehicle mode compared to the walking mode for daily recreation parks. In the same 15 min, 58.6% of the study area and 82.1% of the urban population were covered. However, the accessibility for some residents was still poor. The overall accessibility of weekly recreation parks accessed by motor vehicles was good; 45.3% of the study area and 58.9% of the urban population could access parks in 5 min, while 90.0% of the study area and 96.8% of the population could access parks in 15 min. In terms of the spatial distribution of parks, because of the location of comprehensive parks along the Hun River and special parks, such as the Beiling Park, in Huanggu district, the park accessibility in these regions was better than that in other regions.

## 4. Discussion

### 4.1. Application of the Landscape Pattern Index Method

In terms of landscape patterns, Cai and Feng (2020) [13] provided optimization suggestions for increasing the area of small green patches by assessing the landscape pattern of urban parks in the main urban area of Tianjin. Zhou and Guo (2003) [66] analyzed the spatial structure of green landscapes in the different regions of Tianjin and concluded that there was a certain connection between the comprehensive condition of the city’s overall landscape and the number of small and medium-sized green patches. Xiao et al. (2004) [67] carried out an ecological comprehensive evaluation of different functional areas to evaluate the landscape pattern of green spaces in the WISCO Industrial Zone and obtained ideal results. Nine indicators in the categories of park area, shape and aggregation were selected to assess the different types of parks. This research method is widely used, scientific and reliable and can provide a scientific basis for optimizing the service quality of urban green space systems.

### 4.2. Measurement of Accessibility Evaluation

Yang et al. (2021) [68] evaluated the accessibility of various green space scales in Guangzhou city using the 2FSCA method. The results revealed great differences in green space equality among different administrative regions. Some regions benefited from the surrounding natural environment, so the residents in these communities could enjoy more green space resources; a similar effect was found in this study. Li and Liu (2009) [63] assessed the supply quality of green spaces in Shenyang city and indicated that the accessibility levels of Dadong district, Yuhong district and Hunnan new district were poor. This result could be attributed to the research considering only park areas greater than 2 hectares, which suggested that the accessibility of green spaces was insufficient under the walking mode. Furthermore, the uneven distribution of urban parks in Tiexi district led to accessibility variations. Assessments of the relationship between green space quality and accessibility using CiteSpace software constitute a research hotspot in the metrology research literature on park green space accessibility. The research results reasonably reflected the correlation between green space quality and accessibility.

### 4.3. Explanation of Differences in Park Types

Daily recreation parks (community parks and garden parks) provide platforms for recreational physical activity for residents and are especially vital for an aging Chinese society [69,70]. Weekly recreation parks (comprehensive parks and special parks) not only satisfy residents’ leisure demand but also play a more significant role in promoting urban ecosystem services [71,72]. The results in this study showed that the current distribution of daily recreation parks is far from meeting residents’ practical needs and that the urban ecosystem service capability needs to be improved, as well.

Säumel et al. (2021) found that residents had a high attachment to the local residential greenery but not to their neighbors in the study of Berlin’s eight disadvantaged neighborhoods. The design and management of residential greenery can contribute to more social connections and physical activities in neighborhood [73]. Battisti et al. (2019) applied the Preliminary Assessment Method and the Species-specific Air Quality index to assess the ecosystem service and analyze the socio-demographic characteristics in a Turin neighborhood. For the 50% low-result neighborhoods, they proposed increasing the number and areas of urban green spaces, as well as trees, to improve residents’ well-being [74]. Dadvand et al. (2012) drew the conclusion that high surrounding greenness had a positive influence on newborns in buffers of 100, 250 and 500 m around each maternal place of residence. However, they only considered the linear distance as spatial distance and set the Normalized Difference Vegetation Index as a surrounding greenness factor [75]. Furthermore, some studies indicated that the promotion of availability and accessibility of green spaces vastly contributes to social cohesion, physical and mental health, and an energetic city [76].

### 4.4. Shortages and Suggestions

In this study, we evaluated urban parks in Shenyang city from the two aspects of supply quality and accessibility, but some deficiencies still exist. For example, some studies have demonstrated the importance of public transportation for providing citizens with access to urban parks. Xu and Wang (2020) [44] considered public transportation when studying the accessibility of urban parks in Pu’er city, China. Because the public traffic net was complicated in the central urban area and the associated data volume was vast, public traffic was not considered in this study. Moreover, high-spatial-resolution road congestion data can be difficult to obtain, so we made an ideal hypothesis in this study by setting a unified 30 s intersection waiting time. Varying traffic conditions also have a certain impact on the research results. Additionally, urban park attractiveness influences the choice of urban parks visited by residents. In future studies, influencing factors such as historic culture and tourist preferences could be quantified and considered to allow the research results to more closely represent reality.

In this study, human mobility and dynamic population distribution were not adequately considered in the green space accessibility measurement, as they are difficult to account for in research. Furthermore, there were difficulties in obtaining interannual data and accounting for seasonal differences in winter cities. Guan et al. (2021) determined that notable seasonal variations in park visitor volumes and park service areas existed in all park cases, and the degree of variation differed from park to park [77], which is particularly significant in winter cities such as Shenyang. It is worth mentioning that Xi et al. (2020) found that the dynamic population utilization regression model can better reflect the service level characteristics of park green spaces [78]. However, their study used the population heatmap only in August (31 days) to study the actual service level over a short period, and obtaining interannual data was difficult. Fortunately, research has filled this knowledge gap. Song et al. (2021) leveraged multisource geospatial big data and a modified assessment framework to evaluate the inequality in urban green space exposure for 303 cities in China and attained ideal results [79]. Generally, data acquisition remains a challenge we face, and this aspect needs to be further discussed and improved in the future.

We evaluated urban parks from the two aspects of supply quality and accessibility, which together form the basis of landscape pattern analyses. Based on the research results, the following suggestions are proposed for the construction of urban green space systems. First, compared to areas through which water systems flow, regions where no water systems flow lack sufficient green spaces, in both number and scale. Thus, the construction of green spaces in areas without water systems should be increased to achieve equality for residents. Second, the supply quality of overall urban parks is low. The construction of garden parks and community parks should be promoted in the future planning of park green spaces to improve the overall supply quality. Third, to support recreational activities, daily recreation parks should be arranged more rationally, and the construction of daily recreation parks should be increased to meet resident demands.

## 5. Conclusions

The supply quality of overall urban parks is low in Shenyang city. The supply quality levels of the four analyzed park types can be ranked as follows: garden parks > community parks > comprehensive parks > specific parks. The supply quality levels of garden parks and community parks are significantly higher than those of special parks and comprehensive parks. In terms of accessibility, the results obtained for different travel modes were as follows, from high accessibility to low: motor vehicle > nonmotor vehicle > walking. Additionally, the service efficiency in walking mode is far below resident demand. We subdivided recreational parks into two types and determined that the accessibility of daily recreation parks is poor, while the overall service efficiency of weekly recreation parks is good, apart from a few regions. The study can provide suggestions for the future planning and construction of green spaces in winter cities based on the results obtained by analyzing the supply quality and spatial accessibility of urban parks. At the same time, from the perspective of landscape patterns, research on the service quality of urban parks can provide new ideas for the further study of green space accessibility.

## Figures and Tables

**Table 1:** The quality of urban parks within the Third Ring Road of Shenyang.

Class	Number	Area (Hectare)	Ratio
Comprehensive parks	30	1224.56	57.4
Special parks	15	647.62	30.4
Community parks	64	224.41	10.5
Garden parks	61	35.60	1.7

**Table 2:** The calculation results of landscape pattern indexes.

Class	PD	LPI	LSI	AREA_AM	SHAPE_MN	DIVISION	MESH	SPLIT	AI
Comprehensive parks	1.50	13.93	9.54	118.16	1.73	0.96	67.52	31.40	97.54
Special parks	0.56	15.53	4.91	199.47	1.58	0.97	61.16	34.67	98.46
Community parks	3.48	0.68	10.69	6.15	1.30	0.99	0.64	3282.30	93.44
Garden parks	2.49	0.12	7.71	0.95	1.05	1.00	0.01	130,688.89	88.60
Overall parks	7.07	15.53	14.04	148.19	1.34	0.93	148.19	14.31	97.16

**Table 3:** The evaluation scores of various parks.

Class	First Principal Component	Second Principal Component	Comprehensive Score
Comprehensive parks	−0.1198	−0.3306	−16.64
Special parks	−0.1820	−0.7465	−32.17
Community parks	1.1222	0.1815	73.67
Garden parks	1.8475	0.1824	118.00
Overall parks	−0.5727	0.9019	−9.55

## Data Availability

Not applicable.

## References

[1] Chan C., Yao X. (2008). Air pollution in mega cities in China. Atmos. Environ..

[2] Wu J. (2014). Urban ecology and sustainability: The state-of-the-science and future directions. Landsc. Urban Plan..

[3] Imhoff M., Zhang P., Wolfe R., Bounoua L. (2010). Remote sensing of the urban heat island effect across biomes in the continental USA. Remote Sens. Environ..

[4] Andersson E., Barthel S., Borgström S., Colding J., Elmqvist T., Folke C., Gren A. (2014). Reconnecting cities to the biosphere: Stewardship of green infrastructure and urban ecosystem services. Ambio.

[5] Kambo A., Drogemuller R., Yarlagadda P.K. (2019). Assessing Biophilic Design Elements for ecosystem service attributes–A sub-tropical Australian case. Ecosyst. Serv..

[6] Qiu G., Zou Z., Li X., Li H., Guo Q., Yan C., Tan S. (2017). Experimental studies on the effects of green space and evapotranspiration on urban heat island in a subtropical megacity in China. Habitat Int..

[7] Song Y., Huang B., Cai J., Chen B. (2018). Dynamic assessments of population exposure to urban greenspace using multi-source big data. Sci. Total Environ..

[8] Pathak V., Tripathi B.D., Mishra V.K. (2011). Evaluation of anticipated performance index of some tree species for green belt development to mitigate traffic generated noise. Urban For. Urban Green..

[9] Maas J., Verheij R.A., Groenewegen P.P., De Vries S., Spreeuwenberg P. (2006). Green space, urbanity, and health: How strong is the relation?. J. Epidemiol. Community Health.

[10] Sugiyama T., Leslie E., Giles-Corti B., Owen N. (2008). Associations of neighbourhood greenness with physical and mental health: Do walking, social coherence and local social interaction explain the relationships?. J. Epidemiol. Community Health.

[11] Zhou W., Wang J., Qian Y., Pickett S., Li W., Han L. (2018). The rapid but “invisible” changes in urban greenspace: A comparative study of nine Chinese cities. Sci. Total Environ..

[12] Chen T., Zhao Y., Yang H., Wang G., Mi F. (2021). Recreational services from green space in Beijing: Where supply and demand meet?. Forests.

[13] Cai C.Y., Feng X. (2020). Landscape Pattern of Park Green Spaces in the City Core of Tianjin Base on GIS. J. Chin. Urban For..

[14] Xing L., Tu Y.R., Wang Z.Q. (2020). Optimization of Green Space Landscape Pattern of Small and Medium-Sized Mountain City Parks Based on GIS. J. Anhui Norm. Univ..

[15] Hansen W.G. (1959). How accessibility shapes land use. J. Am. Inst. Planners.

[16] Liu Z., Zhang C., Dai T. (2018). Measuring accessibility of multi-type urban public service facilities with entropy in Beijing. Econ. Geogr..

[17] Zheng Z., Xia H., Ambinakudige S., Qin Y., Li Y., Xie Z., Zhang L., Gu H. (2019). Spatial accessibility to hospitals based on web mapping API: An empirical study in Kaifeng, China. Sustainability.

[18] Jin M., Liu L., Tong D., Gong Y., Liu Y. (2019). Evaluating the spatial accessibility and distribution balance of multi-level medical service facilities. Int. J. Environ. Res. Public Health.

[19] Dejen A., Soni S., Semaw F. (2019). Spatial accessibility analysis of healthcare service centers in Gamo Gofa Zone, Ethiopia through geospatial technique. Remote Sens. Appl. Soc. Environ..

[20] Zhang L., Yin Z., Chen C., Hu H., Liu H., Hao L.I.U. (2020). Accessibility evaluation and layout optimization of urban parks in Xihu District, Nanchang City based on walking of the elderly people. J. Landsc. Res..

[21] Guo S., Song C., Pei T., Liu Y., Ma T., Du Y., Chen J., Fan Z., Tang X., Peng Y. (2019). Accessibility to urban parks for elderly residents: Perspectives from mobile phone data. Landsc. Urban Plan..

[22] Yang H., Zhang X., Li L., Li X., Wang B. (2018). Changing spatial pattern and accessibility of primary and secondary schools in a poor mountainous county: A case study of Song County, Henan Province. Chin. Prog. Geogr..

[23] Cheng Y., Wang J., Rosenberg M.W. (2012). Spatial access to residential care resources in Beijing, China. Int. J. Health Geogr..

[24] Chen Y., Yue W., Rosa D.L. (2020). Which communities have better accessibility to green space? An investigation into environmental inequality using big data. Landsc. Urban Plan..

[25] Wolch J.R., Byrne J., Newell J.P. (2014). Urban green space, public health, and environmental justice: The challenge of making cities ‘just green enough’. Landsc. Urban Plan..

[26] Rigolon A., Browning M., Jennings V. (2018). Inequities in the quality of urban park systems: An environmental justice investigation of cities in the United States. Landsc. Urban Plan..

[27] Wüstemann H., Kalisch D., Kolbe J. (2017). Access to urban green space and environmental inequalities in Germany. Landsc. Urban Plan..

[28] Yu K., Duan T., Li D., Peng J. (1999). Landscape accessibility as a measurement of the function of urban green system. City Plan. Rev..

[29] Hladnik D., Pirnat J. (2011). Urban forestry—Linking naturalness and amenity: The case of Ljubljana, Slovenia. Urban For. Urban Green..

[30] Shi T., Li J., Li Y., Yin H. (2016). Analysis of urban park accessibility in Shenyang City. Chin. J. Ecol..

[31] Neuvonen M., Sievänen T., Tönnes S., Koskela T. (2007). Access to green areas and the frequency of visits—A case study in Helsinki. Urban For. Urban Green..

[32] Niemelä J., Saarela S.R., Söderman T., Kopperoinen L., Yli-Pelkonen V., Väre S., Kotze D.J. (2010). Using the ecosystem services approach for better planning and conservation of urban green spaces: A Finland case study. Biodivers. Conserv..

[33] Song Y.M., Chen B., Kwan M.P. (2020). How does urban expansion impact people’s exposure to green environments? A comparative study of 290 Chinese cities. J. Clean. Prod..

[34] Yin H., Xu J. (2009). Spatial accessibility and equity of parks in Shanghai. Urban Stud..

[35] Nicholls S., Shafer C.S. (2001). Measuring Accessibility and Equity in a Local Park System: The utility of geospatial technologies to park and recreation professionals. J. Park Recreat. Adm..

[36] Kessel A., Green J., Pinder R., Wilkinson P., Grundy C., Lachowycz K. (2009). Multidisciplinary research in public health: A case study of research on access to green space. Public Health.

[37] Silalahi F.E.S., Hidayat F., Dewi R.S., Purwono N., Oktaviani N. (2020). GIS-based approaches on the accessibility of referral hospital using network analysis and the spatial distribution model of the spreading case of COVID-19 in Jakarta, Indonesia. BMC Health Serv. Res..

[38] Zhu Y., Wang C., Jia B., Su J. (2011). GIS-based analysis of the accessibility of urban forests in the central city of Guangzhou, China. Chin. Acta Ecol. Sin..

[39] Ma L., Cao X. (2006). A GIS-based evaluation method for accessibility of urban public green landscape. Acta Sci. Nat. Univ. Sunyatseni.

[40] Alvarez F.D., Madridejos J.M., Sarmiento J.A., Valdez E., Lecaros L.L. (2021). A framework for measuring geospatial amenity accessibility in the Philippines. Int. Arch. Photogramm. Remote Sens. Spat. Inf. Sci..

[41] Jia P., Wang F., Xierali I.M. (2017). Using a Huff-based model to delineate hospital service areas. Prof. Geogr..

[42] Hodgart R.L. (1978). Optimizing Access to public services: A review of problems, models and methods of locating central facilities. Prog. Hum. Geogr..

[43] Lotfi S., Koohsari M.J. (2009). Measuring objective accessibility to neighborhood facilities in the city (A case study: Zone 6 in Tehran, Iran). Cities.

[44] Xu X.D., Wang J. (2020). Study on accessibility optimization of urban recreation green space. Chin. Landsc. Archit..

[45] Nicholls S. (2001). Measuring the accessibility and equity of public parks: A case study using GIS. Manag. Leis..

[46] Guo S., Fan Z.Y., He J.Q., Li Z.L. (2019). On park accessibility in Xixiangtang District of Nanning based on network analysis. Chin. Landsc. Archit..

[47] Tu X., Huang G., Wu J. (2018). Contrary to common observations in the west, urban park access is only weakly related to neighborhood socioeconomic conditions in Beijing, China. Sustainability.

[48] Sun Z.R., Yin H.W., Kong F.H. (2012). Study on Different Calculation Methods of Park Accessibility. China Popul. Resour. Environ..

[49] Zhang J., Fu M., Tao J., Huang Y., Hassani F.P., Bai Z. (2010). Response of ecological storage and conservation to land use transformation: A case study of a mining town in China. Ecol. Model..

[50] Boentje J.P., Blinnikov M.S. (2007). Post-Soviet forest fragmentation and loss in the Green Belt around Moscow, Russia (1991–2001): A remote sensing perspective. Landsc. Urban Plan..

[51] Wu W., Wang Y. (2021). Evaluation and promotion of the service capacity of urban public open spaces based on improving accessibility: A case study of Shenyang City, China. Chin. Geogr. Sci..

[52] Lu N., Li J., Yan H., Shi T., Li Y. (2014). Analysis on accessibility of urban park green space: The case study of Shenyang Tiexi District. Chin. J. Appl. Ecol..

[53] Li L., Dong C. (2019). Research on park layout and accessibility of Nanjing main urban area based on GIS technology. Landsc. Archit..

[54] Li M. (2020). Study on the Accessibility and Service Level of Urban Park Green Space Based on GIS: A Case Study of Yuelu District of Changsha City. Chin. Geomat. World.

[55] Ye T., Zhao N., Yang X., Ouyang Z., Liu X., Chen Q., Hu K., Yue W., Qi J., Li Z. (2019). Improved population mapping for China using remotely sensed and points-of-interest data within a random forests model. Sci. Total Environ..

[56] Qian Y., Zhou W., Yu W., Pickett S.T. (2015). Quantifying spatiotemporal pattern of urban greenspace: New insights from high resolution data. Landsc. Ecol..

[57] Wu W., Wang Y., Liu M., Li C. (2021). A Review on the Use of Landscape Indices to Study the Effects of Three-Dimensional Urban Landscape Patterns on Haze Pollution in China. Pol. J. Environ. Stud..

[58] Zeng H., Chen L.D., Ding S.Y. (2017). Landscape Ecology.

[59] Lu C., Qi W., Li L., Sun Y., Qin T., Wang N. (2012). Applications of 2D and 3D landscape pattern indices in landscape pattern analysis of mountainous area at county level. Chin. J. Appl. Ecol..

[60] Shrestha S., Kazama F. (2007). Assessment of surface water quality using multivariate statistical techniques: A case study of the Fuji river basin, Japan. Environ. Model. Softw..

[61] Deng J., Huang Y., Chen B., Tong C., Liu P., Wang H., Hong Y. (2019). A methodology to monitor urban expansion and green space change using a time series of multi-sensor SPOT and Sentinel-2A images. Remote Sens..

[62] Jolliffe I.T., Cadima J. (2016). Principal component analysis: A review and recent developments. Philosophical transactions of the royal society A: Mathematical. Phys. Eng. Sci..

[63] Li X., Liu C. (2009). Accessibility and service of Shenyang’s urban parks by network analysis. Chin. Acta Ecol. Sin..

[64] Jin Y.F., Gao Y.F., Shen J. (2018). Green space system planning fine regulation—Research on the layout of daily recreational green space. Chin. Landsc. Archit..

[65] Yi Z., Feng L.J., Dong Q.L., Zhang L., Zhang Q.P. (2020). Research on accessibility analysis and layout optimization of park green space based on ArcGIS technology: A case study of Xuchang. Chin. Mod. Urban Res..

[66] Zhou T., Guo D. (2003). GIS-based study on spatial structure of urban greenbelt landscapes: Taking Ningbo City as an example. Chin. Acta Ecol. Sin..

[67] Xiao R., Zhou Z., Wang P., Ye Z., Guo E., Ji G. (2004). Landscape pattern analysis and comprehensive assessment of greenbelt in Wuhan steel & iron industrial district. Chin. Acta Ecol. Sin..

[68] Yang W.Y., Li X., Chen H.L., Cao X.S. (2021). Multi-scale accessibility of green spaces and its equity in Guangzhou based on multi-mode two-step floating catchment area method (M2SFCA). Chin. Acta Ecol. Sin..

[69] Cauwenberg J.V., Cerin E., Timperio A., Salmon J., Deforche B., Veitch J. (2015). Park proximity, quality and recreational physical activity among mid-older aged adults: Moderating effects of individual factors and area of residence. Int. J. Behav. Nutr. Phys. Act..

[70] Cauwenberg J.V., Cerin E., Timperio A., Salmon J., Deforche B., Veitch J. (2017). Is the association between park proximity and recreational physical activity among mid-older aged adults moderated by park quality and neighborhood conditions?. Int. J. Environ. Res. Public Health.

[71] Xie Q., Yue Y., Sun Q., Chen S., Lee S., Kim S. (2019). Assessment of ecosystem service values of urban parks in improving air quality: A case study of Wuhan, China. Sustainability.

[72] Villaseñor N., Escobar M. (2019). Cemeteries and biodiversity conservation in cities: How do landscape and patch-level attributes influence bird diversity in urban park cemeteries?. Urban Ecosyst..

[73] Säumel I., Hogrefe J., Battisti L., Wachtel T., Larcher F. (2021). The healthy green living room at one’s doorstep? Use and perception of residential greenery in Berlin, Germany. Urban For. Urban Green..

[74] Battisti L., Pomatto E., Larcher F. (2019). Assessment and mapping green areas ecosystem services and socio-demographic characteristics in Turin neighborhoods (Italy). Forests.

[75] Dadvand P., Sunyer J., Basagana X., Ballester F., Lertxundi A., Fernandez-Somoano A., Estarlich M., Garcia-Esteban R., Mendez M., Nieuwenhuijsen M. (2012). Surrounding greenness and pregnancy outcomes in four Spanish birth cohorts. Environ. Health Perspect..

[76] World Health Organization (2016). Urban Green Spaces and Health.

[77] Guan C.H., Song J., Keith M., Zhang B., Akiyama Y., Da L.J., Shibasaki R., Sato T. (2021). Seasonal variations of park visitor volume and park service area in Tokyo: A mixed-method approach combining big data and field observations. Urban For. Urban Green..

[78] Song Y.M., Chen B., Ho H.C., Kwan M.P., Liu D., Wang F., Wang J.H., Cai J.X., Li X.J., Xu Y. (2021). Observed inequality in urban greenspace exposure in China. Environ. Int..

[79] Xi J.L., Wu Z.F., Zhang H., Wei J.Y. (2020). Comprehensive evaluation of park green space service capability in central city: Models and cases. Chin. Ecol. Environ. Sci..

